# Prediction of overall survival in primary lung adenocarcinoma using machine learning and deep learning: a cohort study in Latin America country cancer center

**DOI:** 10.3389/fonc.2026.1917778

**Published:** 2026-09-03

**Authors:** Javier H. Gil-Gómez, Carlos Carvajal-Fierro, Ricardo Bruges-Maya, Ixchel Rodríguez Parada, Andrés Mosquera-Zamudio, Julián Riaño-Moreno, José Fernando Polo, Marcela Gómez-Suárez, John Jaime Sprockel, Rafael Parra-Medina

**Affiliations:** 1 Instituto de Investigación, Universidad FUCS, Bogotá, Colombia; 2 Grupo de Investigación CIMET, Universidad de Salamanca, Zamora, Spain; 3 Departamento de Cirugía de Tórax, Instituto Nacional de Cancerología y Fundación CTIC, Bogotá, Colombia; 4 Departamento de Oncología Clínica, Instituto Nacional de Cancerología, Bogotá, Colombia; 5 Clínica Colsanitas, Laboratorio Clínico y de Patología, Bogotá, Colombia, Fundación Universitaria Sanitas, Bogotá, Colombia; 6 Departamento de Patología, Instituto Nacional de Cancerología, Bogotá, Colombia; 7 Universidad Cooperativa de Colombia, Villavicencio, Colombia; 8 Departamento de Patología, Universidad FUCS, Bogotá, Colombia

**Keywords:** deep learning, Latin America, lung cancer, machine learning, survival analysis

## Abstract

**Background:**

Lung adenocarcinoma is the most common subtype of non-small cell lung cancer and a leading cause of cancer-related mortality worldwide. Its marked clinical heterogeneity results in variable outcomes and poor prognosis for many patients. This study aimed to develop and compare statistical, machine learning, and deep learning models for predicting overall survival (OS) in patients with primary lung adenocarcinoma.

**Methods:**

This retrospective cohort study included patients with primary lung adenocarcinoma. Missing data were handled using fold-specific multiple imputation by chained equations within a nested cross-validation framework. Predictor selection and hyperparameter tuning were performed within 5 outer and 3 inner folds using univariable Cox analysis, Elastic Net, XGBoost-based importance, and clinical criteria. Overall survival was modeled using penalized Cox regression, Random Survival Forest, DeepSurv, and DeepHit. Model performance was evaluated from out-of-fold predictions using concordance indices, time-dependent AUC, calibration and the integrated Brier score. Sensitivity analyses included propensity score-matched survival comparisons by biomarker status among patients with stage IV disease.

**Results:**

The cohort included 254 patients, with a median overall survival of 12.8 months. Random Survival Forest showed the best performance (Harrell’s C-index, 0.760; mean time-dependent AUC, 0.851; integrated Brier score, 0.160) and clearly separated high- and low-risk groups (p < 0.001). Clinical stage, ECOG status, systemic treatment, sex, and age were among the most influential predictors. Calibration was acceptable, with slight survival overestimation. Exploratory propensity score-matched analyses in stage IV subgroups suggested the highest apparent performance for RSF, although the small matched samples limited definitive conclusions.

**Conclusions:**

In this cohort, machine learning models showed potential for overall survival prediction in lung adenocarcinoma. RSF achieved the highest observed discrimination, while the penalized Cox model showed competitive performance with lower complexity. These findings suggest that RSF may complement conventional survival models; however, the retrospective single-center design, moderate sample size, missing data, and lack of external validation limit their generalizability.

## Introduction

Lung cancer is one of the most common malignancies worldwide, with approximately 2.21 million new cases diagnosed annually and a five-year mortality rate of around 75% (1). In Latin America and the Caribbean, it is the third most diagnosed cancer, following prostate and breast cancer, with 97,601 new cases reported annually. Despite this ranking, it remains the leading cause of cancer-related mortality, accounting for 86,627 deaths each year (2). Several risk factors have been identified in the development of lung cancer, including active smoking, exposure to environmental tobacco smoke, chronic obstructive pulmonary disease, a family history of lung cancer, infections, and genetic susceptibility (3). Clinical symptoms typically manifest in advanced stages of the disease and may include persistent cough, chest pain, dyspnea, hoarseness, wheezing, unexplained weight loss, and loss of appetite. In many cases, patients also experience generalized fatigue and weakness, as well as recurrent respiratory infections (such as bronchitis or pneumonia) that do not respond to standard treatment (4).

Lung cancer is primarily classified into two major groups: non-small cell lung cancer (NSCLC) and small cell lung cancer (SCLC). NSCLC accounts for approximately 80–85% of cases and is further subdivided into adenocarcinoma (ADC), squamous cell carcinoma, and large cell carcinoma. Adenocarcinoma is the most frequent subtype, particularly among non-smokers and women, and is typically located in the peripheral regions of the lung (5). The common therapeutic approaches include surgery, radiotherapy, chemotherapy, targeted therapies, immunotherapy, local ablative treatments, and palliative care (6).

Survival in lung cancer patients depends on several clinical and biological factors (7), one of the most important is the TNM stage, as it describes tumor size, lymph node involvement, and the presence of metastasis, all of which directly influence disease progression and treatment options (8). Performance status, measured using the Karnofsky or ECOG scales, is also fundamental, as it reflects the patient’s overall condition and ability to tolerate therapy (9). In addition, variables such as sex, age, and the histological and molecular characteristics of the tumor can significantly affect survival, demonstrating that prognosis results from the interaction of multiple factors (10). In the last years, it has also been observed that the survival may change according with the molecular alteration and the target therapy, in fact in the Latin American population with *EGFR* mutation have been shown improved in the overall survival and lower progression risk treated with *EGFR-*TKIs compared to wild-type (WT) patients on chemotherapy (11).

In survival analysis, the Kaplan-Meier estimator and the Cox proportional hazards model are the most widely used classical approaches (12). The Kaplan-Meier method allows for estimating the probability of survival in the presence of censored data and comparing patient groups; however, it is not possible to simultaneously assess the effect of multiple variables (13). The Cox model, on the other hand, allows for analyzing the impact of various covariates on the risk of an event using adjusted hazard ratios, although it assumes of proportionality of risks and a log-linear relationship between covariates and risk—conditions that are not always met in real-world clinical data (14, 15). However, clinical evolution can vary widely among patients, even among those with similar initial characteristics. This heterogeneity supports the use of machine learning-based survival models capable of integrating multiple clinical variables and capturing complex risk patterns over time (16). Given the retrospective design and potential confounding from baseline differences between molecular subgroups, propensity score matching was also performed in stage IV patients as a sensitivity analysis to assess the robustness of biomarker–survival associations. In recent years, several machine learning models have been developed to predict overall survival in lung cancer patients. Among the most widely used approaches are decision trees, neural networks, Bayesian models, gradient boosting algorithms, support vector machines, and clustering methods, among others (17). Machine learning methods are ideal for analyzing highly dimensional data and do not strictly depend on parametric or semiparametric assumptions. This allows them to overcome several limitations of traditional statistical approaches by capturing complex and non-linear relationships between covariates and outcomes, enabling greater predictive power and improved risk stratification in complex clinical contexts (14, 17, 18).

Most machine learning models for lung cancer survival prediction have been developed using datasets predominantly composed of Caucasian or Asian populations (16–18), which may limit their applicability in other contexts and affect the accuracy of predictions, highlighting the need for more representative datasets (19). The objective of our study is to develop and evaluate machine learning and deep learning models to predict overall survival in patients with primary lung adenocarcinoma using a Latin American cohort.

## Materials and methods

**Figure 1:**
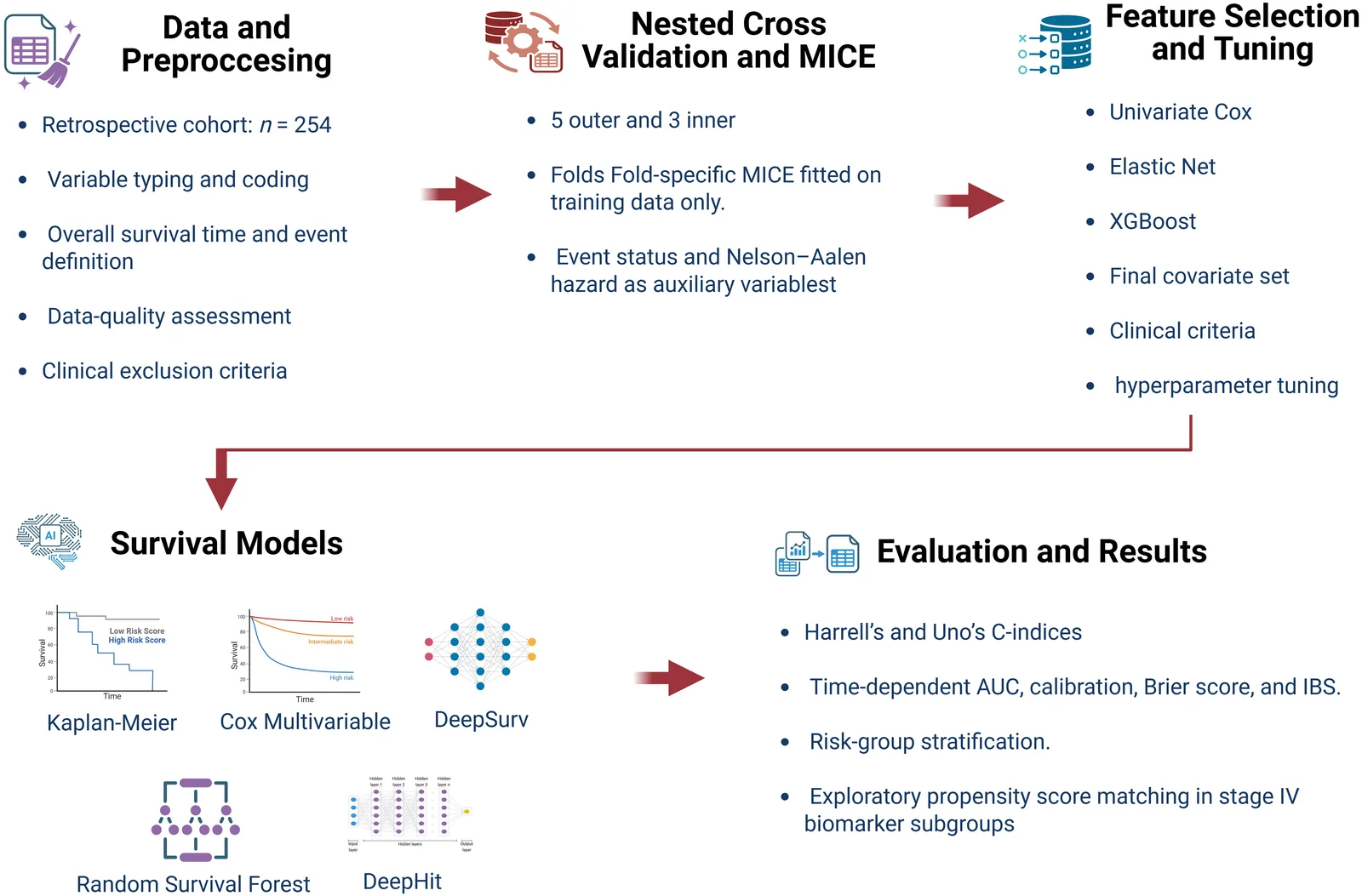
Survival modeling workflow. Created using BioRender.

The project workflow, illustrated in **Figure 1**, included data preprocessing and cleaning, multiple imputations, structured covariate selection, and the development of survival models. Following data cleaning and imputation, stable and clinically relevant predictors were identified and used to compare classical statistical models and machine learning–based approaches. Model performance was evaluated using discrimination and calibration metrics to determine their predictive accuracy and risk stratification capability.

### Dataset

A retrospective cohort study was conducted including patients with histopathologically confirmed primary lung adenocarcinoma diagnosed and treated at the National Cancer Institute of Colombia between May 2010 and October 2025. Clinical and pathological variables were collected for each patient, along with treatment and follow-up information. All patient data were fully anonymized in accordance with the data collection protocol.

Inclusion criteria were patients aged 18 years or older with a histologically confirmed diagnosis of primary pulmonary adenocarcinoma and available clinical information to determine baseline characteristics, survival time, and event status. Cases with missing, contradictory, or chronologically inconsistent dates that prevented a reliable calculation of survival time or classification of event status were excluded.

### Data preprocessing

The initial database was curated by retaining baseline covariates available at the time of diagnosis with less than 18% missing data and established clinical relevance. Data preprocessing included verification of variable consistency, identification of missing values, review of date inconsistencies, and construction of the survival-time and event-status variables.

Multiple imputation was performed independently within each training partition of the nested cross-validation framework using the miceforest package in Python. Twenty imputed datasets were generated, with 15 iterations per imputation. Event status and time-to-event information, represented by the Nelson–Aalen cumulative hazard estimate, were included as auxiliary predictors but were not imputed. The imputation models were fitted exclusively on the training data and subsequently applied to the corresponding validation partition.

### Covariate selection

A total of 14 baseline clinical and molecular covariates were considered as candidate predictors of overall survival. Feature selection was performed independently within each training fold of the nested cross-validation framework. All candidate covariates were evaluated across the fold-specific imputed datasets using four selection criteria: univariable Cox regression, Elastic Net Cox regression at $\lambda_{min}$ and $\lambda_{1SE}$, and XGBoost variable importance.

Univariable Cox regression retained covariates with p < 0.20, Elastic Net retained covariates with non-zero coefficients, and XGBoost-Cox retained covariates with normalized gain equal to or greater than the mean expected importance. A covariate was retained when it met the corresponding criterion in at least 70% of the imputations and was supported by at least three of the four selection criteria.

### Survival models

In this study, predictive models were developed and compared to estimate overall survival (OS) at the individual level. The analysis was not intended to assess causal associations, but rather to determine which model achieved the best prognostic performance, defined by its discrimination, overall prediction error over time, and calibration. The multivariable Cox model was used as the reference approach, against which more flexible machine learning and deep learning methods were compared, including Random Survival Forest (RSF), DeepSurv, and DeepHit.

The Cox proportional hazards regression model is a semi-parametric method commonly used as a benchmark in survival analysis to model the relationship between a set of covariates and the hazard of the event of interest (16).This model assumes that the hazard ratio between individuals remains constant over time (proportional hazards assumption) and defines the conditional hazard function as:


h(t│x)=h_0 (t)  exp(β^T x)

where $h_{0}\;(t)$represents the unspecified baseline hazard function, x is the vector of covariates, and β is the vector of coefficients estimated through partial likelihood maximization. Another implemented model was RSF, a non-parametric tree-based ensemble method designed to analyze right-censored survival data (15). Unlike the Cox model, RSF does not assume proportional hazards and can capture nonlinear relationships and complex interactions among covariates. Similarly, DeepSurv and DeepHit incorporate neural networks to extend the survival modeling framework. DeepSurv retains the proportional hazards assumption of the Cox model but replaces the linear predictor with a nonlinear function learned by a neural network (20). In contrast, DeepHit adopts a more flexible approach by directly modeling the time-to-event distribution without assuming proportional hazards (15). The inclusion of these approaches enabled the comparison of classical statistical, machine learning, and deep learning models within a unified predictive framework. All models were evaluated using nested cross-validation, with five outer folds for independent performance assessment and three inner folds for feature selection and hyperparameter tuning. Within each outer fold, the models were trained on each of the 20 imputed training datasets. Patient-level predictions were then averaged across imputations to obtain a single out-of-fold prediction for each patient. Performance metrics were calculated from the complete set of out-of-fold predictions.

Model performance was assessed using standard survival analysis metrics, with Harrell’s C-index and Uno’s C-index as the primary measures of discrimination, the Integrated Brier Score (IBS) as a measure of overall prediction error over time, and the time-dependent area under the receiver operating characteristic curve (AUC(t)) to evaluate discrimination at different time horizons while accounting for censored data.

The calibration of the penalized Cox, RSF, DeepSurv, and DeepHit models was assessed at 12, 24, and 36 months using out-of-external skinfold survival predictions. At each horizon, the observed survival status was weighted using inverse probability censoring weighting (IPCW), with censoring distributions estimated solely from the corresponding out-of-external skinfold. Overall calibration and calibration slope were estimated using an IPCW-weighted logistic calibration model, with ideal values of 0 and 1, respectively. Flexible IPCW calibration curves and the Integrated Calibration Index (ICI) were also calculated. Uncertainty was quantified using patient-level bootstrap 95% confidence intervals stratified by skinfold.

### Survival analysis

To estimate time to death and characterize associated prognostic factors, overall survival was analyzed in a cohort of patients with primary lung adenocarcinoma, followed between May 2010 and October 2025. Survival time was defined as the interval between the date of diagnosis and death from any cause. Patients who were still alive at the time of the administrative cutoff or who lacked information after their last recorded visit were considered censored observations. Overall survival was initially estimated using the Kaplan-Meier method, which provides a non-parametric estimate of the survival function and allows for the description of the time-to-event distribution in the cohort. This analysis constitutes an empirical reference for the subsequent evaluation of the performance of predictive survival models (13). Differences in survival between clinically and molecularly relevant subgroups, including clinical stage, functional status and molecular profile— were assessed using the log-rank test, which allows testing the null hypothesis of equality between the survival curves of the groups being compared (12, 13).

### Sensitivity analysis using propensity score matching

To account for potential baseline imbalances between molecular subgroups in this retrospective cohort, exploratory propensity score matching was applied among patients with stage IV disease. Separate matched comparisons were performed for the EGFR-positive versus EGFR-negative, ALK-positive versus ALK-negative, and PD-L1 ≥1% versus <1% subgroups. Propensity scores were estimated using baseline clinical covariates, including age, sex, pooled ECOG performance status, personal history of cancer, history of lung disease, and smoking history. Matching was performed independently across the 20 imputed datasets using 1:1 nearest neighbor matching without replacement and a caliber of 0.20 on the propensity score logit. The balance of covariates before and after matching was assessed using standardized mean differences, considering values ​​ less than 0.20. Post-matching survival was assessed using Kaplan-Meier estimates, probabilities of survival at 12, 24 and 36 months, and restricted mean survival time (RMST).

## Results

### Data selected

The final analytical cohort comprised 254 patients with lung adenocarcinoma, including 160 deaths (62.99%) and 94 censored observations (37.01%), as summarized in **Table 1**. Advanced disease was frequent, with 61.02% of patients diagnosed at clinical stage IV. Women represented 61.02% of the study population. Most patients had an ECOG performance status of 0–1 (64.56%), followed by ECOG 2–3 (30.31%). A history of smoking was reported in 40.94% of patients, while 26.38% had a personal history of cancer. Regarding the molecular profile, EGFR mutations were identified in 29.66% of the evaluated cases and ALK rearrangements in 13.39%, whereas PD-L1 expression below 1% was the most frequent category (48.03%).

**Table 1:** Clinical, demographic, and biomarker characteristics of patients with primary lung adenocarcinoma (n = 254).

Characteristics	n (n:254)	%
Median Age (IQR)	67 (57–73 years)	
Gender		
Male	99	38,98%
Female	155	61,02%
Personal history of cancer		
Yes	67	26,38%
No	173	68,11%
Missing data	14	5.51%
Family history of lung cancer		
Yes	13	5,12%
No	199	78,35%
Missing data	42	16,54%
History of lung disease		
Yes	44	17,32%
No	194	76,38%
Missing data	16	6,30%
Baseline functional status		
ECOG 0	60	23,62%
ECOG 1	104	40,94%
ECOG 2	57	22,44%
ECOG 3	20	7,87%
ECOG 4	1	0,39%
Missing data	12	4,72%
Smoking history		
Yes	104	40,94%
No	128	50,39%
Missing data	22	8,66%
Metastasis		
Yes	169	66,53%
No	82	32,28%
Missing data	3	1,18
Clinical stage of the disease		
I	50	0,1969
II	6	0,0236
III	18	0,0709
IV	155	0,6102
Missing data	25	0,0984
EGFR mutation		
Absent	154	0,6063
Present	75	0,2966
Missing data	25	0,0984
ALK rearrangements		
Absent	183	0,7205
Present	34	0,1339
Missing data	37	0,1457
PD-L1 measurement		
Less than 1%	122	48.03%
Between 1 and 50%	70	0,2756
Greater than 50%	17	0,0669
Missing data	45	0,1772
Systemic treatment with palliative intent		
Yes	122	48,03
No	95	37,4
Missing data	37	14,57
Received palliative radiotherapy		
Yes	66	0,2598
No	151	59,45
Missing data	37	0,1457
Overall survival status		
Events (Deaths)	160	0,6299
Censored	94	0,3701

ALK, Anaplastic Lymphoma Kinase; EGFR, Epidermal Growth Factor Receptor; ECOG, Eastern Cooperative Oncology Group. PD-L1, Programmed Death-Ligand 1; IQR, interquartile range.

### Covariate selection

Fourteen candidate covariates were selected: age, sex, personal history of cancer, family history of lung cancer, history of lung disease, baseline ECOG performance status, smoking history, clinical stage (TNM), EGFR mutation, ALK rearrangement, PD-L1 expression, systemic treatment with palliative intent, extrathoracic therapy, and metastases. Receipt of maintenance therapy after first-line treatment, second-line palliative treatment, and pack-year index were excluded because their missing-data rates exceeded 50%, which affected model stability. Variable selection was performed independently within each training partition; therefore, the set of selected predictors could vary across partitions. Selection frequencies are presented in **Supplementary Figure 1**.

### Performance of survival models

Model performance was estimated from out-of-fold predictions generated through nested cross-validation, with predictions averaged across 20 imputations within each outer fold (**Table 2**). RSF achieved the highest discriminative performance across all agreement and discrimination metrics, including Harrell’s C-index, Uno’s C-index, and time-dependent AUC, demonstrating a superior ability to classify patients by survival risk. The Cox model ranked second in discrimination and showed good calibration. DeepHit was competitive in discrimination but had weaker calibration. DeepSurv, despite having the lowest discriminative performance, achieved the second-lowest IBS, demonstrating a trade-off between the two dimensions. Overall, RSF outperformed the other models in discrimination and calibration, while Cox remained competitive, particularly in calibration.

**Table 2:** Survival model performance.

Model	Concordance		Discrimination	Prediction error
	Harrell C-index	Uno C-index	Mean AUC(t)	Mean IBS
Penalized Cox	0.745 (0.719–0.778)	0.737 (0.705–0.769)	0.814 (0.769–0.861)	0.171 (0.155–0.186)
Random Survival Forest	0.760 (0.732–0.796)	0.752 (0.719–0.792)	0.851 (0.816–0.893)	0.160 (0.144–0.175)
DeepSurv	0.705 (0.676–0.745)	0.700 (0.669–0.739)	0.790 (0.746–0.838)	0.192 (0.178–0.206)
DeepHit	0.522 (0.469–0.573)	0.562 (0.521–0.611)	0.561 (0.503–0.631)	0.314 (0.290–0.343)

Values are point estimates (95% bootstrap confidence intervals) from pooled out-of-fold predictions generated using 5-fold outer and 3-fold inner nested cross-validation. AUC(t), time-dependent area under the curve; IBS, integrated Brier score.

Permutation importance analysis of the RSF model identified clinical stage (TNM), initial functional status (ECOG), and systemic management with palliative intent as the variables with the greatest impact on individual risk prediction, as illustrated in **Supplementary Figure 2**. More advanced clinical stages and greater functional status deterioration were consistently associated with an increase in predicted risk, consistent with reported clinical evidence for advanced lung cancer (3, 7, 8). Likewise, patients who received palliative systemic treatment had a lower predicted risk compared to those who did not receive it. This finding could reflect both the possible beneficial effect of treatment and the fact that treated patients tend to have better baseline clinical status; however, due to the observational nature of the study, it is not possible to establish a direct causal relationship between these variables and survival.

**Figure 2:**
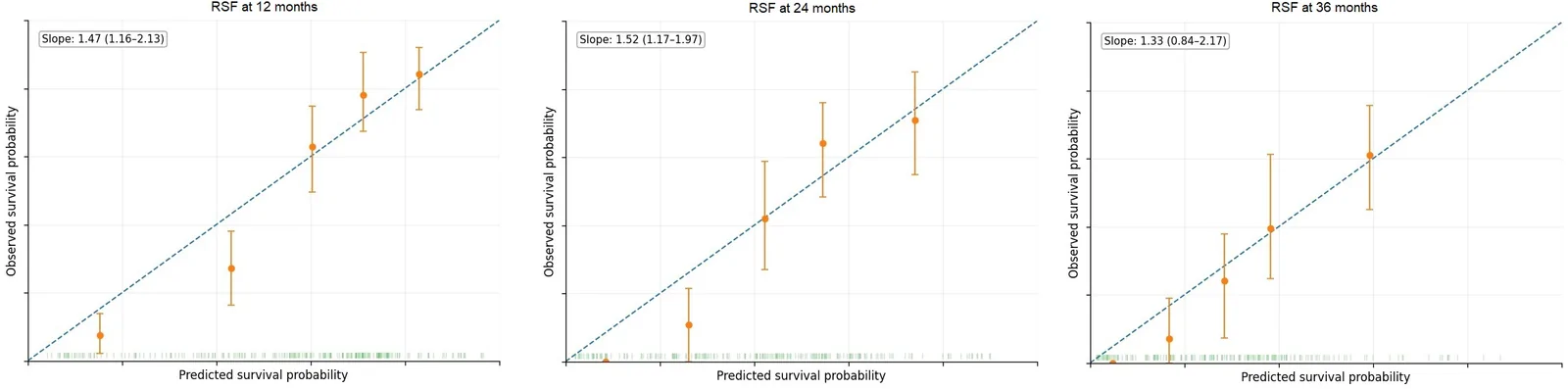
Calibration of RSF survival predictions.

Regarding calibration, the RSF calibration slopes were 1.47 (95% CI: 1.16–2.13), 1.52 (95% CI: 1.17–1.97), and 1.33 (95% CI: 0.84–2.17) at 12, 24, and 36 months, respectively. Estimates were more precise at 12 and 24 months, whereas the wider confidence interval at 36 months indicated greater uncertainty (**Figure 2**). Calibration results for all evaluated models are presented in **Supplementary Figure 3**.

### Survival analysis

**Figure 3:**
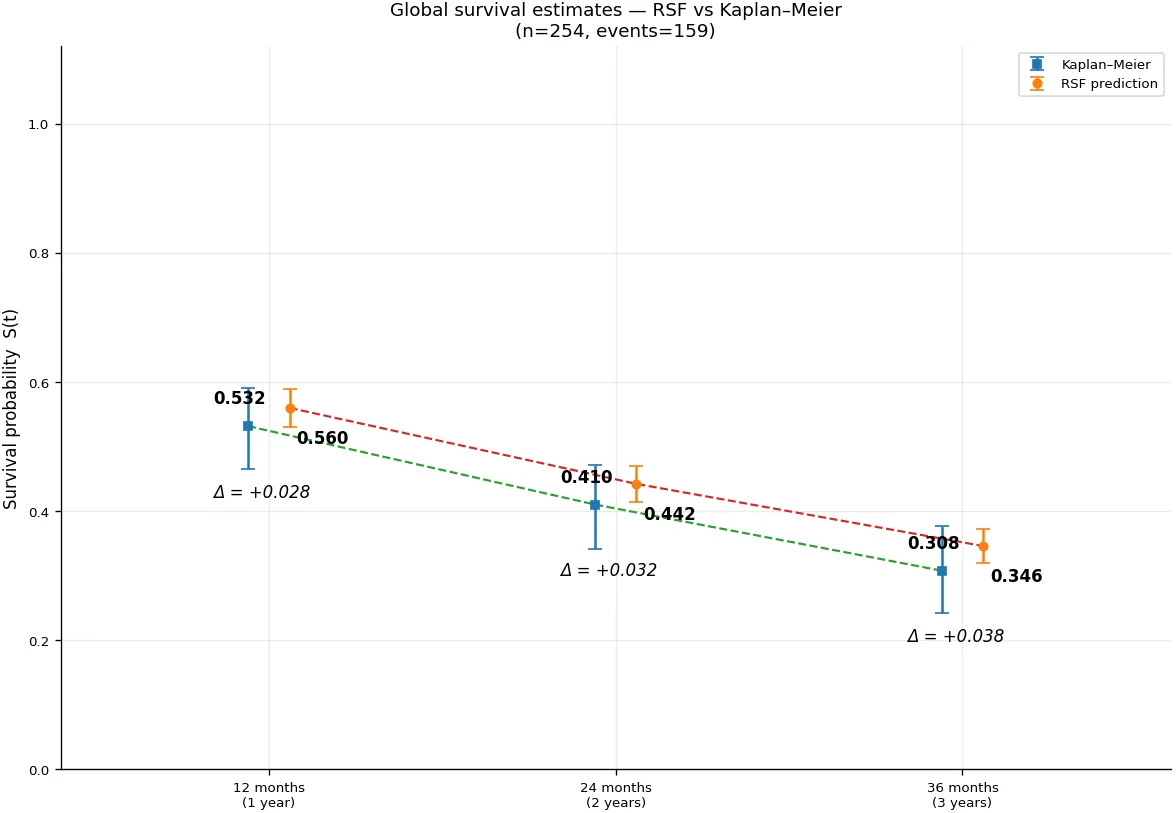
Comparison of RSF-predicted survival and Kaplan–Meier estimates.

Overall survival was evaluated in a final cohort of 254 patients. During follow-up, 160 patients (62.99%) experienced the event of interest, defined as death from any cause, whereas 94 patients (37.01%) were censored, indicating that they did not present the event during the observation period. The Kaplan–Meier analysis estimated an overall survival probability of 53.2% at one year, 41.0% at two years, and 30.8% at three years, with a median survival of 12.8 months. The median follow-up of 30 months supports the reliability of survival estimates up to the 36-month time horizon. The RSF model estimated survival probabilities of 56.0%, 44.2%, and 34.6% at 12, 24, and 36 months, respectively. The absolute differences from the Kaplan–Meier estimates ranged from 0.028 to 0.038, indicating a close overall correspondence between the model-based and observed survival estimates (**Figure 3**).

Kaplan-Meier curves revealed prognostic differences according to clinical stage, baseline ECOG performance status, and sex. Stage IV comprised the largest patient group (n = 155) and showed an early decline in survival. The number of patients remaining at risk decreased from 155 at baseline to 64, 32, and 11 at 12, 24, and 36 months, respectively. Survival differed significantly across clinical stages (log-rank p < 0.001). In contrast, patients with stage I disease (n = 50) showed the most favorable survival pattern, with 23 patients remaining at risk at 24 months (**Supplementary Figure 4A**). Similarly, patients with poorer baseline ECOG performance status showed lower survival. At 24 months, 27 of 60 patients with ECOG 0 remained at risk, whereas no patients with ECOG 3–4 (n = 21) remained at risk (**Supplementary Figure 4B**). When stratified by sex, women (n = 155) showed higher survival than men (n = 99). At 24 months, 48 women and 18 men remained at risk, respectively (log-rank p = 0.001; **Supplementary Figure 4C**). Risk stratification based on the RSF-predicted risk score separated the cohort into two prognostic groups. Patients classified as high risk (n = 107) showed a steeper decline in survival, with 25, 11, and 4 patients remaining at risk at 12, 24, and 36 months, respectively. In contrast, the low-risk group (n = 147) showed a more favorable survival pattern, with 89, 55, and 26 patients remaining at risk at the same time points. The survival curves differed significantly between groups (log-rank p < 0.001). This separation indicates a strong discriminative ability of the RSF-derived risk score, with significantly poorer survival among high-risk patients compared with low-risk patients (log-rank p < 0.001; **Supplementary Figure 4D**).

### Sensitivity analysis

Propensity score matching (PSM) was successfully integrated into the 20 imputed sets for *EGFR*, *ALK*, and PD-L1 in stage IV patients. After propensity score matching, adequate covariate balance was achieved across the matched cohorts. The mean standardized mean difference (SMD) values were 0.0751, 0.0670, and 0.0459, with an average of 45.65, 23.95, and 50.35 matched pairs for the *EGFR, ALK*, and PD-L1 analyses, respectively.

In the post-PSM survival analysis, *EGFR* was the biomarker with the most consistent pattern. *EGFR-positive* patients had a longer median survival than *EGFR-negative* patients (13.74 vs. 5.50 months) and a higher 12-month survival rate (59.8% vs. 34.9%), with an absolute difference of 24.9 percentage points (95% CI: 2.5 to 47.4; p = 0.029). Furthermore, the 12-month RMST was 2.54 months longer in the *EGFR-positive* group (95% CI: 0.48 to 4.59; p = 0.016). Exploratory RMST estimates suggested differences at 24 months, whereas estimates at later horizons were less precise. For ALK and PD-L1, the positive groups showed slightly longer median survival; however, the small matched samples and wide confidence intervals did not provide clear evidence of differences in survival, RMST, or robust post-PSM Cox models. For ALK, the small matched sample size (approximately 24 pairs) and the width of the confidence intervals limited the precision of the estimates. Therefore, these results should be considered exploratory and hypothesis-generating only, and do not allow for firm inferential conclusions. Full results by biomarker are presented in **Supplementary Figures 5**–**S7**. In the matched datasets, RSF yielded the highest C-index (0.7718), followed closely by the multivariable Cox model (0.7617). Each imputed matched dataset included approximately 92 patients. Given the modest sample size and the small difference between models, these exploratory findings suggest broadly comparable discrimination rather than clear superiority of RSF. DeepSurv showed intermediate performance, whereas DeepHit had the lowest performance and the greatest variability across imputations and folds. Additional performance metrics are reported in **Supplementary Table 1**.

## Discussion

In the present study, the Cox proportional hazards model was compared with several machine learning and deep learning approaches for predicting overall survival in patients with primary lung adenocarcinoma from a Latin American cohort. To the best of our knowledge, this is one of the first studies to develop and evaluate machine learning and deep learning models for overall survival using exclusively data from Latin American patients. This population represents a unique and highly heterogeneous demographic group shaped by complex patterns of genetic admixture as well as distinct social determinants of health. In the context of lung cancer, this diverse genetic ancestry has been shown to influence disease susceptibility, molecular tumor profiles, and clinical outcomes (21–23). It has been described the “Hispanic paradox”, characterized by lower mortality rates from NSCLC despite disproportionately lower socioeconomic status and reduced access to healthcare services (24, 25). Notably, foreign-born Hispanics demonstrate a significantly reduced risk of disease-specific mortality compared with their U.S.-born counterparts (hazard ratio [HR] 0.90; 95% confidence interval (CI), (0.87–0.96)) (26). Several mechanistic hypotheses have been advanced to explain this paradox, including epigenetic models proposing that environmental exposures, such as dietary patterns, cultural practices, and social determinants of health, may modulate gene expression through reversible epigenetic modifications, thereby influencing cancer susceptibility, tumor biology, and survival outcomes (27).

We observed that the RSF model demonstrated the best performance in terms of discrimination, achieving the highest values for the C-index and time-dependent AUC, outperforming the multivariable Cox model and neural network-based approaches. While the Cox model showed good overall calibration according to IBS, its discriminative capacity was lower than that of the RSF. The selected predictor variables—including age, sex, TNM stage, ECOG performance status, and variables related to therapeutic management—are consistent with the most frequently described prognostic factors in the literature, supporting their clinical relevance and value in risk estimation (7, 8, 10, 28, 29). TNM stage, ECOG performance status, systemic treatment, sex, and age were identified as the most influential predictors in the RSF model, a finding consistent with that reported in previous studies (15–19). Regarding missing-data handling, previous studies have relied on single-imputation approaches, such as mean or mode imputation (30, 31), or alternative machine-learning methods, including MissForest (15). In contrast, our study implemented fold-specific multiple imputation by chained equations within the nested cross-validation framework. Imputation models were fitted exclusively within the corresponding training partitions, using event status and the training-fold Nelson–Aalen cumulative hazard as auxiliary variables. This strategy preserved the relationship between the predictors and survival outcomes while minimizing information leakage. Predictions were subsequently averaged across the imputed datasets. By accounting for the uncertainty associated with missing values, MICE reduce the risk of biased estimates and supports more reliable confidence intervals (32), thereby strengthening model development and evaluation.

The performance of RSF as the model with the greatest discriminatory power in our study (C-index: 0.77) is consistent with trends reported in previous studies, where RSF outperformed Cox and DeepHit (33, 34). In larger cohorts, this advantage appears to increase, with reported C-index values of 0.85 in 50,687 patients and 0.739 in 17,322 patients (30, 35). However, this relationship is not uniform, as Cox outperformed RSF in one study (0.90 vs. 0.86) (36), while DeepSurv was identified as the best-performing model in a cohort closer in size to ours (n = 471), although with a lower C-index than that observed in our study for all evaluated models (37). The narrow variation between models in our study (range 0.74–0.77) is also in line with patterns reported in comparable cohorts (34, 38). Several machine learning studies applied to lung cancer have addressed survival prediction as a binary classification problem, transforming the outcome into events such as “12- or 24-month survival” (16–19, 30). Although this approach allows the use of traditional classification models, it does not explicitly model the time-to-event nature of survival or adequately incorporate censoring, both fundamental elements in survival analysis (7, 12, 13). Furthermore, most of these studies have been developed using widely available public databases, such as SEER or TCGA, or hospital cohorts from Asia or Europe (8–10, 15–19).

DeepHit showed substantially lower discrimination and higher prediction error than the Cox and RSF models. Although DeepHit can model nonlinear relationships and time-dependent effects without assuming proportional hazards, these theoretical advantages did not translate into improved performance in this cohort (12, 13, 16). Its lower performance may be related to the limited sample size, the presence of missing data, and the smaller effective training sets generated within each cross-validation fold. Deep learning survival models generally require larger datasets and extensive hyperparameter optimization to learn stable temporal patterns and avoid overfitting (16–19).

Although models as DeepSurv and DeepHit have shown outstanding results in scenarios with unstructured data, such as medical images or genomic sequences (15, 39) their performance was lower in the present study. Moreover, the predominantly tabular structure of the covariates may have favored the penalized Cox model and RSF, which are often more suitable for moderate-sized clinical datasets. Therefore, the observed performance likely reflects the interaction between model complexity, sample size, and data structure rather than an inherent limitation of DeepHit (17, 19, 39, 40). From a calibration perspective, the RSF preserved the expected ordering of risk groups, but the slopes above 1 at the earlier horizons suggest that predicted survival probabilities were somewhat compressed relative to the observed outcomes. Although the slope at 36 months was closer to the ideal value, this finding should not be interpreted as improved long-term calibration because the estimate was less precise. Given the median overall survival of approximately 13 months, fewer patients remained at risk at 36 months, increasing uncertainty at this horizon. Thus, the model maintained risk ordering, but agreement between predicted and observed probabilities remained imperfect, particularly for longer-term predictions (15, 18, 39).

Survival analyses suggested prognostic heterogeneity among patients with lung adenocarcinoma, supporting the exploration of multivariable and machine learning approaches to characterize complex risk patterns over time (12, 16, 18). Propensity score matching was performed as an exploratory sensitivity analysis in stage IV subgroups with improved covariate balance, without the intention of establishing causal effects (41). In these matched samples, RSF showed the highest apparent predictive performance, closely followed by the multivariable Cox model; however, the limited sample size precludes definitive conclusions regarding differences between the models. EGFR positivity showed a possible early survival advantage, particularly at 12 months, but this association attenuated at later time horizons (42). Findings for ALK and PD-L1 were also inconclusive because of the small matched subgroups and wide confidence intervals. Therefore, these results should be considered preliminary and hypothesis-generating. Future studies should include larger prospective multicenter cohorts, adequate representation of each biomarker subgroup, comprehensive adjustment for treatment-related factors, and external validation to determine whether these patterns are reproducible and clinically relevant.

The main limitations of this study include its retrospective, single-center design and moderate sample size, which may have constrained the performance of data-intensive deep learning models such as DeepSurv and DeepHit. Moreover, because of the retrospective nature of the data, variables were not consistently recorded at the same clinical time point, including diagnosis, completion of staging, treatment decision, and treatment initiation. This temporal heterogeneity may have introduced measurement variability and limited the comparability of predictors across patients. The predominance of advanced-stage disease may also have influenced the distribution of events and survival estimates (28, 29) Differences in access to targeted therapies and the substantial proportion of patients receiving palliative care could have influenced the observed outcomes (28, 29, 43). Maintenance therapy after first-line treatment and second-line palliative therapy were excluded because they represented post-baseline exposures available only to patients who survived long enough to receive them, thereby introducing a risk of immortal-time bias. In addition, both variables had more than 50% missing data, and inconsistent recording of treatment dates prevented reliable landmark or time-dependent analyses. Most patients lacked a complete baseline molecular profile, limiting the identification and evaluation of candidates for targeted therapy. These findings should therefore be regarded as a reference for future studies rather than definitive evidence. Subsequent research should use larger and more representative cohorts, apply more comprehensive and clinically informed covariate selection, define a uniform and clinically meaningful prediction time point for all patients, and conduct adequately powered propensity score analyses with sufficient covariate balance and overlap. A more detailed evaluation of treatment type, timing, sequencing, and response will also be required to better distinguish baseline prognostic factors from treatment-related effects and reduce potential time-dependent biases.

## Conclusions

In this cohort of patients with lung adenocarcinoma, the evaluated models showed potential for overall survival prediction. Random Survival Forest achieved the highest observed discrimination, whereas the penalized Cox model provided competitive performance with lower complexity. However, the modest sample size, missing data, retrospective design, and lack of external validation limit the generalizability and immediate clinical applicability of these findings. Machine learning methods may complement conventional survival models, but validation in larger, independent, and preferably prospective multicenter cohorts is required before their use in clinical prognostic stratification.

## Data Availability

The datasets are available from the corresponding author upon reasonable request, subject to ethical and confidentiality restrictions.

## Supplementary material

Supplementary Figure 1 Frequency of covariate selection among nested folds and selection methods.

Supplementary Figure 2 Comparison of RSF-predicted survival and Kaplan–Meier estimates.

Supplementary Figure 3 Calibration of survival models.

Supplementary Figure 4 **(A–D)** Kaplan–Meier survival by selected baseline covariates.

Supplementary Figure 5 Post-PSM Kaplan–Meier survival by EGFR status.

Supplementary Figure 6 Post-PSM Kaplan–Meier survival by ALK status.

Supplementary Figure 7 Post-PSM Kaplan–Meier survival by PDL1 status.

Supplementary Table 1 Comparative performance of post-PSM survival models.

Supplementary Table 2 Architectures, Hyperparameters, and Tuning Procedures Model.
