# Supplementary material for: Prediction of overall survival in primary lung adenocarcinoma using machine learning and deep learning: a cohort study in Latin America country cancer center
Source: Front Oncol. 2026 Sep 3;16:1917778. doi: 10.3389/fonc.2026.1917778 (PMC13581495; doi:10.3389/fonc.2026.1917778)
Supplement: Supplementary file 1 [file SupplementaryFile1.docx]

**Supplementary Information: Prediction of Overall Survival in Primary Lung Adenocarcinoma Using Machine Learning and Deep Learning: A Cohort Study at a Low- and Middle-Income Country Cancer Center.**

This Supplementary Information includes Figures S1–S8 and Tables S1–S2.

**Supplementary Figure S1.** Frequency of nested covariate selection across outer cross-validation folds. Mean selection frequencies are shown for univariable Cox, Elastic Net Cox using $\lambda_{min}$, Elastic Net Cox using $\lambda_{1SE}$, and XGBoost-Cox. The dashed vertical line indicates the predefined 70% selection-frequency threshold; covariates selected at or above this threshold were considered consistently selected

**Supplementary Figure S2.**  Random Survival Forest permutation importance across the outer validation folds of nested cross-validation. Variable importance was quantified as the mean decrease in Uno’s C-index after permuting each predictor. Within each outer fold, multiple imputation and model fitting were performed using training data only, with results averaged across 20 imputed datasets. Larger positive values indicate a greater contribution to predictive performance, whereas values near or below zero indicate limited predictive contribution

.

**Supplementary Figure S3.** **Nested out-of-fold calibration of survival predictions at 12, 24, and 36 months.** Calibration plots are shown for penalized Cox regression, DeepSurv, and DeepHit using predictions obtained from the outer folds of the nested cross-validation. The dashed diagonal represents perfect calibration, while orange points and error bars indicate grouped observed survival estimates with 95% confidence intervals. Green rug marks show the distribution of predicted survival probabilities. Calibration slopes with 95% confidence intervals are displayed in each panel.

**Supplementary Figure S4.** Kaplan–Meier survival according to selected baseline covariates and RSF-predicted risk groups. Survival curves are presented by (A) clinical stage, (B) baseline ECOG performance status, (C) sex, and (D) high versus low risk derived from Random Survival Forest out-of-fold predictions. Differences between groups were assessed using the log-rank test. Numbers at risk at 0, 12, 24, and 36 months are shown below each panel.

**Sensitivity analysis**

In the survival analysis following propensity score matching, the most relevant finding was observed in the comparison by EGFR. On average, 45.65 imputation-matched pairs were included, corresponding to 91.30 patients and 69.15 events. EGFR-positive patients had a longer median survival than EGFR-negative patients (13.74 vs. 5.50 months). At 12 months, survival was higher in the EGFR-positive group (59.8% vs. 34.9%), with an absolute difference of 24.9 percentage points (95% CI: 2.5 to 47.4; p = 0.029). Consistently, the 12-month RMST was 2.54 months longer in EGFR-positive patients (95% CI: 0.48 to 4.59; p = 0.016). At 24 months, the RMST difference also favored EGFR-positive patients (4.22 months; 95% CI: 0.03 to 8.40; p = 0.048), whereas survival differences at later horizons were less precise.

**Supplementary Figure S5.** Kaplan–Meier estimates of overall survival according to EGFR mutation status after propensity score matching. Survival curves compare EGFR-positive and EGFR-negative patients in the representative imputed matched dataset. Shaded areas represent 95% confidence intervals. The lower table shows the number of patients at risk, censored observations, and cumulative events at each time point. Vertical dashed lines indicate 12-, 24-, and 36-month landmarks.

For ALK, the post-PSM analysis included an average of 23.95 imputation-matched pairs, corresponding to 47.90 patients and 29.50 events. Although ALK-positive patients had a slightly longer median survival than ALK-negative patients (21.94 vs. 18.04 months), the difference was not statistically significant. At 12 months, survival was 69.1% in ALK-positive patients versus 60.5% in ALK-negative patients, an absolute difference of 8.7 percentage points (95% CI: −30.4 to 47.7; p = 0.664). No significant differences were observed in 24- or 36-month survival or in RMST at the assessed time horizons. The robust Cox proportional hazards model post-PSM did not show a significant association between a positive ALK test and overall survival (HR = 1.109; 95% CI: 0.445–2.759; p = 0.825). These results are inconclusive due to the small matched sample size and the width of the confidence intervals.

**Supplementary Figure S6.** Kaplan–Meier estimates of overall survival according to ALK rearrangement status after propensity score matching. Survival curves compare ALK-positive and ALK-negative patients in the representative imputed matched dataset. Shaded areas represent 95% confidence intervals. The lower table displays the number of patients at risk, censored observations, and cumulative events across follow-up. Vertical dashed lines indicate 12-, 24-, and 36-month landmarks.

In the post-PSM analysis for PD-L1, an average of 50.35 imputation-matched pairs were included, corresponding to 100.70 patients and 72.30 events. Patients with PD-L1 ≥1% had a longer median survival than those with PD-L1 <1% (12.96 vs. 9.76 months); however, the differences were not statistically significant. At 12 months, survival was 54.2% in the PD-L1 ≥1% group versus 46.2% in the PD-L1 <1% group, with an absolute difference of 8.0 percentage points (95% CI: −10.2 to 26.2; p = 0.387). No significant differences were observed at 24 or 36 months, nor in the RMST.

**Supplementary Figure S7.** Kaplan–Meier estimates of overall survival according to PD-L1 expression after propensity score matching. Survival curves compare patients according to PD-L1 expression status in the representative imputed matched dataset. Shaded areas represent 95% confidence intervals. The lower table reports the number of patients at risk, censored observations, and cumulative events at each time point. Vertical dashed lines indicate 12-, 24-, and 36-month landmarks

|  | **Concordance indices** | | **Predictive performance** | |
| --- | --- | --- | --- | --- |
| **Model** | **C-index (Harrell)** | **C-index (Uno)** | **Integrated Brier Score (IBS)** | **Mean AUC(t)** |
| **Multivariable Cox** | 0.7617 ± 0.0268 | 0.7370 ± 0.0256 | 0.1733 ± 0.0138 | 0.8273 ± 0.0387 |
| **Random Survival Forest** | 0.7718 ± 0.0250 | 0.7518 ± 0.0224 | 0.1709 ± 0.0163 | 0.8610 ± 0.0299 |
| **DeepSurv** | 0.7088 ± 0.1157 | 0.7002 ± 0.1110 | 0.1673 ± 0.0481 | 0.7867 ± 0.1768 |
| **DeepHit** | 0.6258 ± 0.1129 | 0.6230 ± 0.1087 | 0.2858 ± 0.0389 | 0.6845 ± 0.1632 |

**Supplementary Table S1** Comparative performance of post-PSM survival models. Validation metrics are reported as mean ± standard deviation. Random Survival Forest achieved the highest discrimination, whereas DeepSurv showed the lowest integrated Brier score. Lower IBS values indicate better prediction accuracy.

| **Model** | **Architecture** | **Hyperparameter search space** | **Tuning procedure** | **Software** |
| --- | --- | --- | --- | --- |
| **Penalized multivariable Cox** | Linear proportional-hazards model with ridge (L2) regularization; Breslow tie handling; standardized continuous predictors and one-hot encoded categorical predictors. | Ridge α: 0.0001–10 (16 prespecified values) Maximum iterations: 1,000 Tolerance: 1×10⁻⁹ | All α values evaluated in the inner CV. Selection maximized the stability-adjusted 36-month Uno C-index. Final models were refitted in each imputed outer-training set. | scikit-survival 0.25.0 scikit-learn 1.7.2 |
| **Random Survival Forest** | Bootstrap ensemble of survival trees; 500 trees during tuning and 1,500 trees for final fitting. | max_features: sqrt, 0.33, 0.50, 0.75 min_samples_leaf: 3, 5, 10, 15 min_samples_split: max(6, 2 × leaf) max_depth: none, 6, 10 max_samples: 0.70, 0.85, none | Reproducible random search of 40 configurations. Hyperparameters selected within the inner CV using the stability-adjusted 36-month Uno C-index. | scikit-survival 0.25.0 scikit-learn 1.7.2 |
| **DeepSurv** | Fully connected neural network with one log-risk output and Cox partial-likelihood loss; optional batch normalization and dropout. | Hidden units: (8), (12), (16), (16–8), (24–12) Dropout: 0–0.35 Learning rate: 1×10⁻⁴–1×10⁻³ Weight decay: 1×10⁻⁵–1×10⁻² Batch normalization: no/yes | Random search of 16 configurations with 2 seeds. AdamW, gradient clipping at 5.0, early stopping (patience 20), ≤160 tuning epochs; final epochs capped at 180. | PyTorch 2.11.0+cu128 |
| **DeepHit** | Single-risk DeepHit with a torchtuples MLPVanilla network and discrete-time output. | Hidden units: (8), (12), (16), (16–8), (24–12) Dropout: 0–0.35 Learning rate: 1×10⁻⁴–1×10⁻³ Batch size: 32, 64 Time intervals: 18, 24, 36 α: 0.60, 0.75, 0.90; σ: 0.10, 0.20, 0.50 | Random search of 16 configurations with 2 seeds. Adam optimization, early stopping (patience 20), ≤160 tuning epochs; final epochs capped at 180. | pycox 0.3.0 torchtuples 0.2.2 PyTorch 2.11.0+cu128 |
| **Shared pipeline** | Data processing, MICE imputation, feature selection, and supporting analyses. | — | Applied within the same nested cross-validation framework. | Python 3.12.13 NumPy 2.0.2 pandas 2.2.2 miceforest 5.7.0 LightGBM 4.5.0 lifelines 0.30.0 XGBoost 3.3.0 |

**Supplementary Table S2. Model Architectures, Hyperparameters, and Tuning Procedures.** Hyperparameters were tuned in 3-fold inner loops within 5-fold nested cross-validation
